# Dual-AAV split prime editor corrects the mutation and phenotype in mice with inherited retinal degeneration

**DOI:** 10.1038/s41392-022-01234-1

**Published:** 2023-02-06

**Authors:** Kaiqin She, Yi Liu, Qinyu Zhao, Xiu Jin, Yiliu Yang, Jing Su, Ruiting Li, Li Song, Jianlu Xiao, Shaohua Yao, Fang Lu, Yuquan Wei, Yang Yang

**Affiliations:** 1 Department of Ophthalmology, West China Hospital, Sichuan University, Chengdu, Sichuan China; 2 State Key Laboratory of Biotherapy and Cancer Center, West China Hospital, Sichuan University and Collaborative Innovation Center, Chengdu, Sichuan China

**Keywords:** Molecular medicine, Gene therapy

## Abstract

The prime editor (PE) can edit genomes with almost any intended changes, including all 12 possible types of base substitutions, small insertions and deletions, and their combinations, without the requirement for double strand breaks or exogenous donor templates. PE demonstrates the possibility of correcting a variety of disease-causing mutations and might expand the therapeutic application of gene editing. In this study, PE was optimized based on a dual-adeno-associated virus (AAV) split-intein system in vitro by screening different split sites and split inteins. We found that splitting PE before amino acid 1105(Ser) of SpCas9 with Rma intein resulted in the highest on-target editing. The orientations of pegRNA and nicking sgRNA in the AAV vector were further optimized. To test the in vivo performance of the optimized dual-AAV split-PE3, it was delivered by subretinal injection in *rd12* mice with inherited retinal disease Leber congenital amaurosis. The prime editors corrected the pathogenic mutation with up to 16% efficiency in a precise way, with no detectable off-target edits, restored RPE65 expression, rescued retinal and visual function, and preserved photoceptors. Our findings establish a framework for the preclinical development of PE and motivate further testing of PE for the treatment of inherited retinal diseases caused by various mutations.

## Introduction

Inherited retinal disease (IRD) is the major cause of blindness globally and is characterized by degeneration of photoreceptors and/or retinal pigment epithelium (RPE). It is a group of heterogeneous disorders associated with mutations in over 250 genes.^1^ The inheritance pattern of this group of disorders varies. Despite extraordinary progress achieved in the development of therapies for these diseases over the last decade, including approved gene therapy for RPE65-related Leber congenital amaurosis (LCA) and current clinical trials for certain recessive IRDs, the majority of IRDs remain incurable.^2^ The development of gene editing techniques based on clustered regularly interspaced short palindromic repeats (CRISPR) has made it possible to correct a wide range of genetic mutations.

Prime editor (PE) is a new CRISPR-based tool that can engineer precise genome edits without the need for a double strand break (DSB) or exogenous donor DNA template.^3^ PE2 is composed of a catalytically impaired SpCas9 nickase (H840A) and an engineered reverse transcriptase (RT). Then PE2 forms a complex with a prime-editing guide RNA (pegRNA) that carries a single-guide RNA (sgRNA) and a 3′ extension containing the RT template with the desired edit and the primer binding site (PBS). PE3 consists of PE2, pegRNA and a nicking sgRNA that induce a nick in the non-edited strand to enhance the prime-editing activity. PE can edit genomes with almost any changes, including all 12 types of base substitutions, small insertions and deletions, and their combinations. The capacity to precisely correct pathogenic mutations independent of their makeup makes PE a very promising approach for the treatment of IRDs, which have a wide mutation spectrum.^4^

Many efforts on PE itself have been made to improve the editing efficiency of PE.^5,6^ However, we believe that optimizing the delivery route of PE to improve in vivo efficiency is another option. Meanwhile, the safe and efficient delivery of PE in vivo is a major issue before its clinical application. Adeno-associated virus (AAV) is the most often utilized vehicle for the in vivo delivery of genetic material due to its good safety profile. However, its limited packaging capacity (~5 kb) is insufficient for the massive PE construct (~6.3 kb). To deliver PE with AAV, we used a split-intein strategy in which PE is split into amino-terminal (N-terminal) and carboxy-terminal (C-terminal) components, which are subsequently reassembled into full-length PE by a *trans-splicing* intein.^7^

In this study, we improved the editing efficiency of the split-intein PE system by screening split inteins and split sites in vitro. We investigated the impact of pegRNA and nicking sgRNA orientations on the editing efficiency of the optimized split PE. To test the in vivo editing efficiency, we integrated the optimized split PE into dual-AAV vectors to target the nonsense mutation of the *Rpe65* gene in *rd12* mice, a mouse model of LCA. We showed that the subretinal delivery of dual-AAV split-PE corrected the pathogenic mutation with up to 16% efficiency in a precise way, with no detectable off-target edits, and restored RPE65 expression, rescued retinal and visual function, and preserved photoceptors.

## Results

### Screening of split-PE for efficient PE delivery

**Fig. 1:**
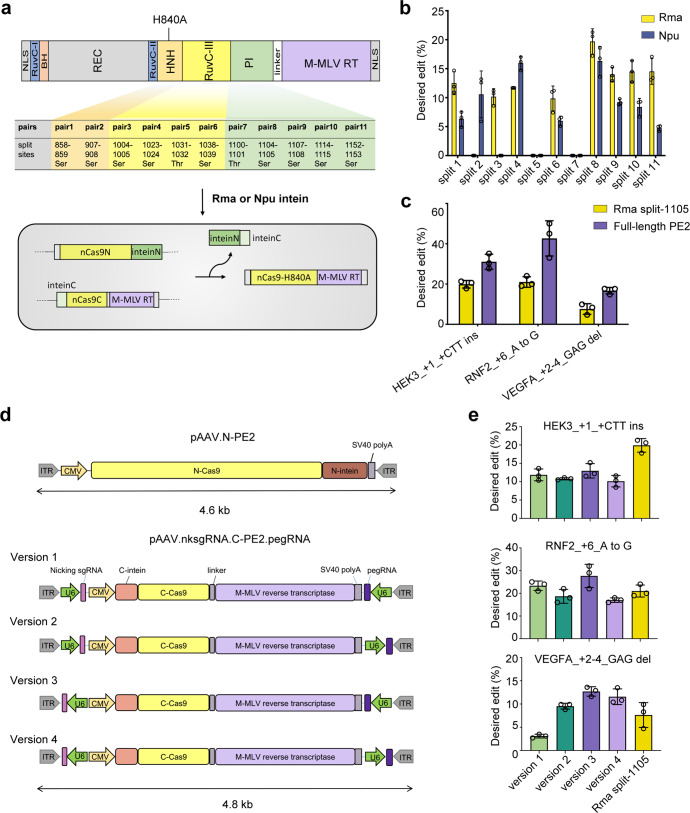
Optimization of split-intein prime editor 3 AAV vectors. **a** Schematic showing split-PEs split at 11 different sites and Rma or Npu intein-mediated PE reconstitution through protein *trans-splicing*. Split pairs 1 and 2 are in the HNH domain, pairs 3 to 6 are in the RuvCIII domain, and pairs 7–11 are in the PI domain. RuvC, endonuclease domain; BH, bridge helix; REC, recognition domain; HNH, HNH endonuclease domain; PI, protospacer-adjacent motif (PAM)-interacting domain; M-MLV RT, engineered Moloney murine leukemia virus (M-MLV) reverse transcriptase. **b** The frequencies of desired editing introduced by different split pairs were quantified by Sanger sequencing and analyzed by EditR. HEK293 cells were transfected with split PE plasmids at equal molar concentrations, pegRNA and sgRNA. Data are shown as mean ± SEM, *n* = 3. **c** Prime-editing efficiencies at three endogenous loci in HEK293 cells transfected with Rma split-1105 or full-length PE2 at equal molar concentrations, pegRNA and sgRNA. Data are shown as mean ± SEM, *n* = 3. **d** Schematic of the N-termin of PE3 and four versions of the C-terminal of PE3 with different orientations of pegRNA and nicking sgRNA. **e** Prime-editing efficiencies at three endogenous loci in HEK293 cells transfected with the N-terminal of PE3 and different versions of the C-terminal of PE3 compared to those in cells transfected with plasmids expressing Rma split-1105, pegRNA and nicking sgRNA. Data are expressed as mean ± SEM, *n* = 3

The choice of split site affects editing efficiency by affecting the reconstitution activity of PE2.^8^ To ensure that the N- and C-terminals were under AAV packaging size restriction, we designed split sites of PE2 within the range from amino acids 855 (Val) to 1155 (Lys) of SpCas9. To achieve high splicing efficiency, cysteine, serine, or threonine residues should follow the C-intein in the C-terminal half of PE.^8,9^ Considering these two conditions, we finally divided PE2 into two halves at 11 different splitting sites (Fig. 1a). Npu intein from *Nostoc punctiforme* DnaE and Rma intein from *Rhodothermus marinus* DnaB, two of the most commonly used inteins in split-Cas9,^10^ were employed in this study. The N-terminals were fused to the N-intein of Npu or Rma, whereas the C-terminals were fused to the C-intein of Npu or Rma (Fig. 1a).

We assessed the editing efficiency of different split-PE pairs for the creation of an insertion at the HEK3 locus (HEK3_+1_CTT ins). HEK293 cells were transfected with different split-PE2 pairs, pegRNA and nicking sgRNA (PE3 strategy, which is used in all experiments in this study). Genomic DNA was isolated, and the desired editing frequencies were quantified. The results showed that Rma outperformed Npu intein in most split sites and dividing PE2 just before the amino acid 1105(Ser) of SpCas9 with the Rma intein (hereafter referred to as the Rma split-1105 construct) resulted in the highest on-target editing (Fig. 1b). Full-length PE2 expression after transfection with Rma split-1105 was observed (Supplementary Fig. S1). Next, the comparison of editing efficiency between Rma split-1105 and full-length PE2 was made in HEK293 cells at three endogenous loci, including base substitution (RNF2_6_GtoA), insertion (HEK3_+1_CTT ins), and deletion (VEGFA_2–4_del). The results showed that the Rma split-1105 editing efficiency was 64.0% of full-length PE2 at HEK3 (19.9% vs 31.1%), 49.2% of full-length PE2 at RNF2 (21.0% vs 42.7%), and 45.5% of full-length PE2 at VEGFA (7.6% vs 16.7%) (Fig. 1c).

To deliver PE3 in dual AAVs, we further cloned the pegRNA and nicking sgRNA into the C-terminal of the PE2 vector (pAAV.nksgRNA.N-PE2.pegRNA) due to the size constraints, with pegRNA and nicking sgRNA flanking the C-terminal of PE2 (Fig. 1d). The efficiency of sgRNA transcription could be affected by the proximity and orientation in relation to inverted terminal repeats (ITRs) of AAV.^11,12^ Four versions of the pAAV.nksgRNA.N-PE2.pegRNA vector were constructed, with the different orientations of the pegRNA and nicking sgRNA expression cassettes within the AAV genome (pAAV.nksgRNA.N-PE2.pegRNA_v1/v2/v3/v4) (Fig. 1d). Cotransfection of pAAV.N-PE2 and each version of pAAV.nksgRNA.N-PE2.pegRNA to HEK293 cells targeting three endogenous loci showed that version 3, in which the expression cassettes of pegRNA and nicking sgRNA were in the reverse orientation of the PE2 C-terminal, was the most efficient among the four versions (Fig. 1e). We also observed an improvement in prime editing at 2 loci (RNF2 and VEGFA) when pegRNA and nicking sgRNA were constructed in line with the C-terminal of PE2, which might be due to the co-expression of pegRNA and nicking sgRNA in the same cell.

### In vitro screening of PE3 for *Rpe65* mutation

**Fig. 2:**
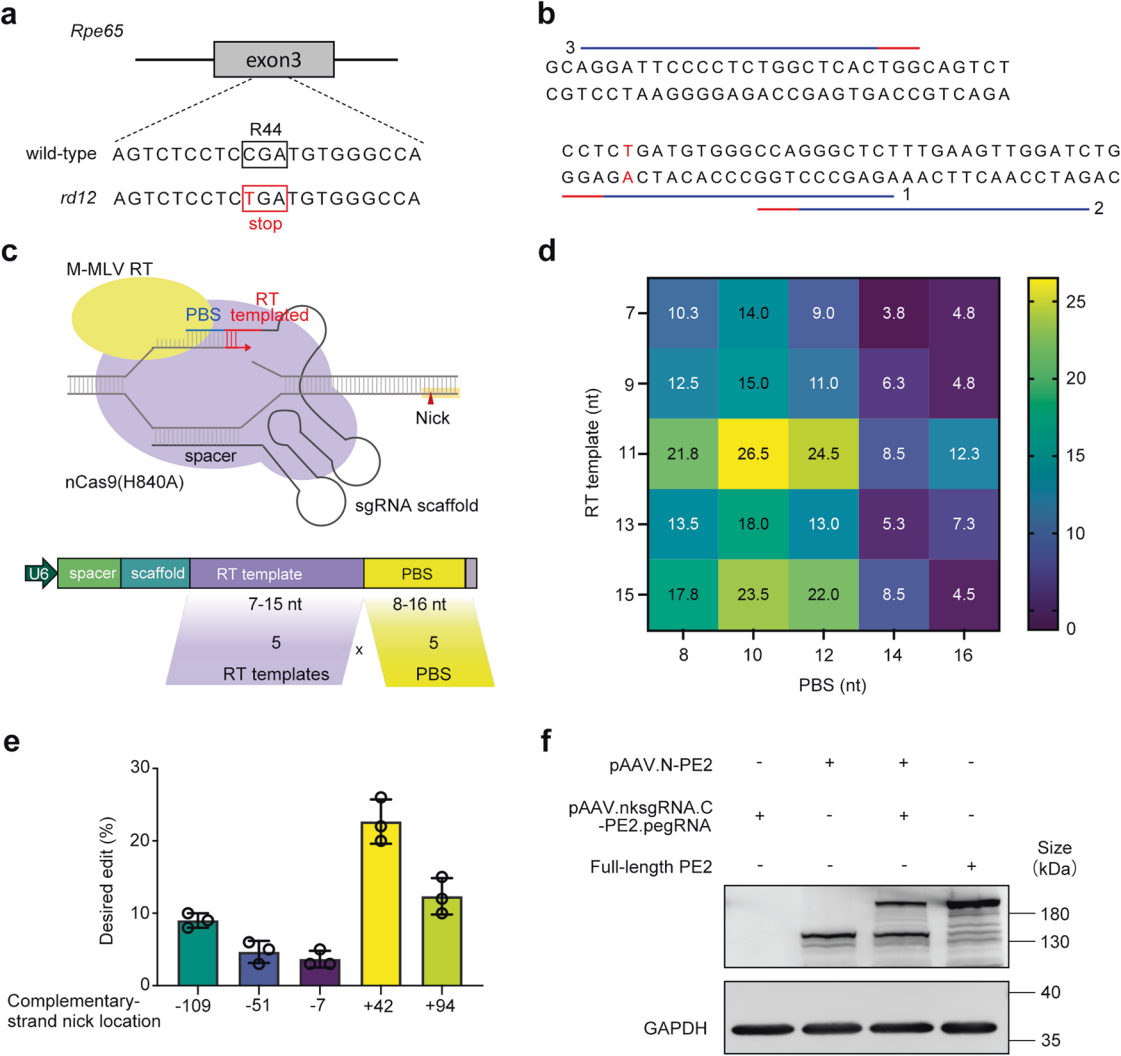
In vitro evaluations of pegRNAs and nicking sgRNAs for *Rpe65* mutation. **a** The *rd12* mouse model has a homozygous C-to-T nonsense mutation (red) in exon 3 of the *Rpe65* gene, resulting in a premature stop codon (red box). **b** PE2 target sequences for correction of the disease-causing point mutation in *Rpe65*. The protospacer and PAM are shown as blue and red lines, respectively. The numbers indicate the target sequence IDs. **c** Diagram of the PE system and schematic of pegRNA screening for the *Rpe65* mutation. A total of 25 pegRNAs containing PBSs of five different lengths (8, 10, 12, 14 or 16 nt) and five different RT template lengths (7, 9, 11, 13 or 15 nt) were evaluated. **d** Comparison of editing performance of different pegRNAs co-transfected with full-length PE2 and nicking sgRNA at +42. Data are expressed as mean, *n* = 3. **e** Comparison of editing performance of nicking sgRNAs at −109, −51, −7, +42, and +94, co-transfected with full-length PE2 and pegRNA containing 10-nt PBS and 11-nt RT template. Data are expressed as mean ± SEM, *n* = 3. **f** Western blot analysis to detect full-length PE2 expression in cells co-transfected with p.AAV.N-PE2 and p.AAV.nksgRNA.C-PE2.pegRNA. GAPDH (36 kDa) was used as a loading control

The above results established an in vivo PE3 delivery solution. However, for each site, a number of other factors (spacer, PBS length, relative position of the alternate strand nick, etc.) affect the editing efficiency. *rd12* is a mouse model of type 2 LCA harboring a nonsense mutation in the *Rpe65* gene on exon 3 (c.130C>T; p.R44X) (Fig. 2a). To screen for the most efficient PE3 for *Rpe65* mutation in vitro, by employing CRISPR/Cas9 to permanently integrate the *rd12* mutant *Rpe65* sequence into the AAVS1 genomic locus, a HEK293-*rd12* mutant cell line was created (Supplementary Fig. S2).

Then, we sought PE2 target sequences near the mutation and three target sequences were found (Fig. 2b). In vitro experiments showed that sequence 1 induced the highest indel frequency (Supplementary Fig.S3) and thus was chosen to be the spacer of pegRNA. Next, pegRNAs containing PBS of five different lengths (8, 10, 12, 14 or 16 nucleotides (nt)) and RT templates of five different lengths (7, 9, 11, 13 or 15 nt) were designed, resulting in 25 pegRNAs (Fig. 2c) (Supplementary Table S2). We evaluated these pegRNAs in HEK293-*rd12* cells and the results showed that pegRNA-11 containing 10-nt PBS and 11-nt RT template produced the highest A to G replacement frequency (26.5%) (Fig. 2d). Then, we designed 5 nicking sgRNAs (Supplementary Table S2). The nicking sgRNA at +42 produced the highest substitution frequency (Fig. 2e). The pegRNA-11 and +42 nicking sgRNA were chosen for the in vivo experiments and cloned into pAAV.nksgRNA.N-PE2.pegRNA. Full-length PE2 expression by pAAV.N-PE2 and pAAV.nksgRNA.N-PE2.pegRNA was confirmed by western blotting (Fig. 2f).

### Subretinal delivery of dual-AAV8 split-PE3 corrects the *Rpe65* mutation efficiently and precisely in vivo

**Fig. 3:**
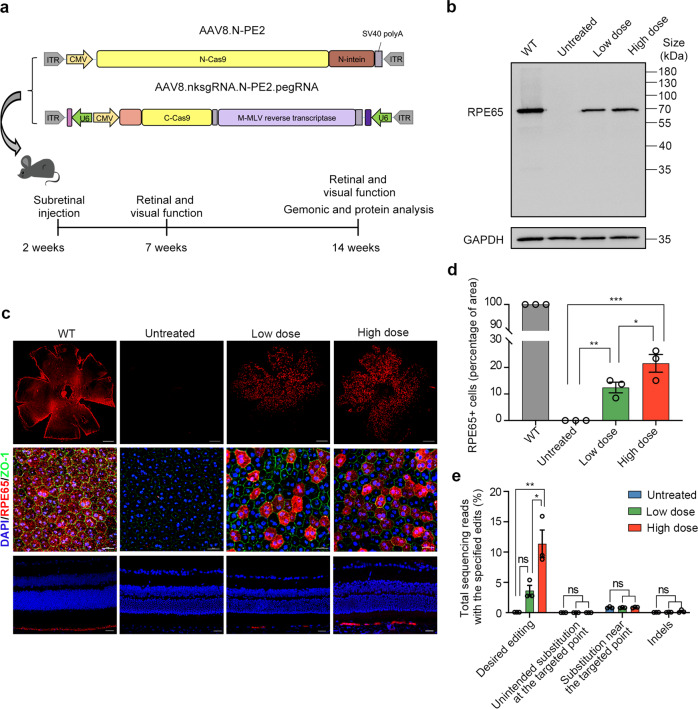
Subretinal delivery of dual-AAV8 split-PE3 corrects mutation and restores expression of *Rpe65* in *rd12* mice. **a** Schematic of the AAV vector genomes for subretinal delivery (top) and timeline of the experimental setup (bottom). Two-week-old *rd12* mice were subretinally injected with a total of 3 × 10^9^ or 1 × 10^10^ GC/eye AAV8 (with the two vectors at 1:1 ratio), referred to as the low dose and high doses, respectively. After 5 and 12 weeks, retinal and visual function were assessed. After 12 weeks, the RPE tissue was harvested for genomic and protein analysis. **b** Western blot analysis to detect RPE65 (65 kDa) expression in mouse RPE tissue lysate 12 weeks post injection. GAPDH (36 kDa) was used as a loading control. **c** Immunofluorescence analysis of representative RPE flatmounts (first row in low magnification and second row in high magnification) and eye cross sections (third row) of treated mice. Red indicates RPE65, blue indicates DAPI, and green indicates ZO-1 (a protein marker for tight junctions). Scale bars, 500 μm for flatmounts in low magnification; 25 μm for flatmounts in high magnification and eye cross sections. **d** Quantification of RPE65^+^ cells from the low magnification images (*n* = 3 in each group) of the RPE flatmounts based on immunofluorescence. Data are shown as mean ± SEM. One-way ANOVA with *post-hoc* Tukey’s multiple comparison tests. *p < 0.05, **p < 0.01, ***p < 0.001. **e** Frequencies of desired and unintended edits in the *rd12* locus in genomic DNA isolated from the RPE tissue of untreated, low-dose-treated, and high-dose-treated eyes (*n* = 3 in each group). Data are shown as mean ± SEM. A 40-bp region centered on the pegRNA nicking site was used to assess substitutions near the targeted nucleotide. A 100-bp region centered on the pegRNA nicking site was used to assess indels. Data are mean ± SEM. One-way ANOVA with *post-hoc* Tukey’s multiple comparison tests. *p < 0.05, **p < 0.01, ns nonsignificant difference

Integrating the above developments, we packaged the optimized expression sequence into dual-AAV8 split-PE3 vectors (AAV8.nksgRNA.N-PE2.pegRNA and AAV8.C-PE2) (Fig. 3a) and injected the two AAVs at a 1:1 ratio subretinally into 2-week-old *rd12* mice. Initially, three doses (a total of 3 × 10^9^, 1 × 10^10^ or 3 × 10^10^ genome copies (GC) per eye) were given. However, the ERG analysis at 5 weeks post injection demonstrated that 3 × 10^10^ GC/eye led to significantly decreased b- and a-wave amplitudes compared to 1 × 10^10^ GC/eye (Supplementary Fig. S4), which might be related to the ocular toxicity of the high AAV dose.^13^ Thus, 3 × 10^9^ and 1 × 10^10^ GC/eye were used in further studies and referred to as the low and high doses, respectively.

The restoration of RPE65 protein in treated eyes was assessed by western blotting and immunofluorescence at 12 weeks post injection. The RPE65 bands were observed in the RPE/choroid/sclera lysates from both the low- and high-dose groups (Fig. 3b). In contrast, no RPE65 protein was detected in untreated eyes. To determine the percentage of corrected cells in each group, RPE flatmounts were stained with an RPE65-specific antibody. The RPE65^+^ area was quantified in the low magnification images (first row of Fig. 3c), which revealed a rescue of 12.2 ± 1.9% in low-dose-treated eyes and 21.6 ± 3.3% in high-dose-treated eyes (Fig. 3d). The correction rate might be underestimated since the transfected area of RPE is approximately 66.9 ± 4.0% (Supplementary Fig. S5). The normal morphology of the corrected RPE was confirmed by high-magnification images of RPE flatmounts and the right layer of the rescued RPE was confirmed by cross sections (Fig. 3c).

To estimate the correction efficiency, we carried out amplicon deep sequencing of RPE tissue. The precise edit was 3.6 ± 0.9% in low-dose-treated eyes and 11.4 ± 2.3% in high-dose-treated eyes, with a maximum correction of up to 5.4% in low-dose eyes and 15.9% in high-dose eyes (Fig. 3e). The reason that the frequency of editing at the DNA level was lower than the frequency of RPE65^+^ cells would be because the DNA samples of RPE cells were mixed with those of cells from the choroid and sclera, which were not exposed to the virus. No substantial unintended substitutions at or near the pegRNA-targeted nucleotide were observed in any of the treated mice compared with the untreated group (Fig. 3e and Supplementary Fig. S6). There was no significant difference in the indel frequency between the low (0.07 ± 0.02%) or high (0.22 ± 0.14%) dose group and the untreated group (0.05 ± 0.01%) (Fig. 3e).

**Fig. 4:**
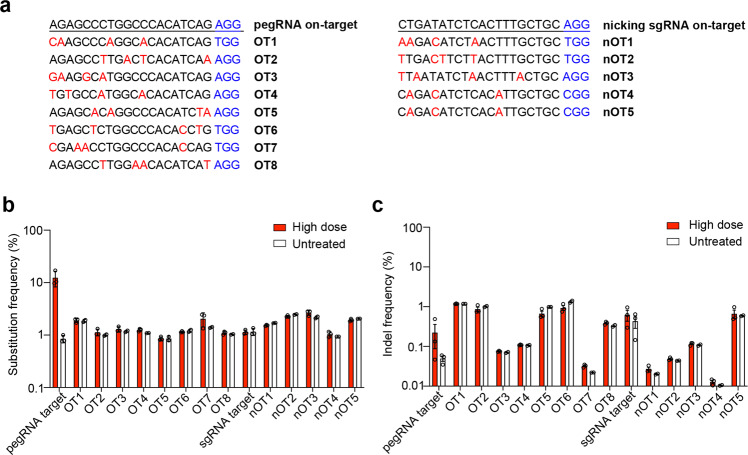
PE3 corrects the pathogenic mutation in a highly precise manner. **a** Potential off-target sites of pegRNA and nicking sgRNA computationally predicted by CRISPOR. Red indicates mismatched sequences, while blue represents PAM sequences. Frequencies of the substitution (**b**) and indels (**c**) at the eight potential off-target sites of pegRNA (OT1–OT8) and at the five potential off-target sites for nicking sgRNA (nOT1–nOT5) in RPE tissues of untreated (*n* = 2–3) and high-dose treated (*n* = 3) *rd12* mice. A 40-bp region centered on the pegRNA nicking site was used to assess substitutions near the targeted nucleotide. A 100-bp region centered on the pegRNA nicking site was used to assess indels. Data are mean ± SEM

We used CRISPOR to identify the top eight and five possible off-target sites of pegRNA and nicking sgRNA, respectively, to quantify the off-target effects^14^ (Fig. 4a). No off-target editing, including substitution and indels, above the background level of the untreated eyes was detected (Fig. 4b, c).

### Prime-editing improves retinal and visual function in *rd12* mice

RPE65 is a critical component in the enzymatic pathway necessary to produce 11-*cis* retinal, the chromophore for rod and cone opsins. The loss of RPE65 results in the disturbance of rod and cone function,^15^ with the profoundly diminished ERG response observed as early as 3 weeks of age.^16^ Cones degenerate rapidly at approximately 2 weeks of age, and by 5 weeks of age, S-opsin-positive cones degenerate extensively, with only M-opsin-positive cones remaining in the peripheral dorsal and temporal regions.^17^

To determine whether the partial restoration of RPE65 expression can rescue rod and cone function, we assessed retinal cell activity by full-field scotopic (rod-derived) and photopic (cone-derived) electroretinography (ERG). Since the mouse has two types of cones, green-sensitive M-cones and UV-sensitive S-cones, photopic ERG was performed using two distinct wavelengths of light. The response of M-cones was stimulated by green light and the response of S-cones was stimulated by UV light.

**Fig. 5:**
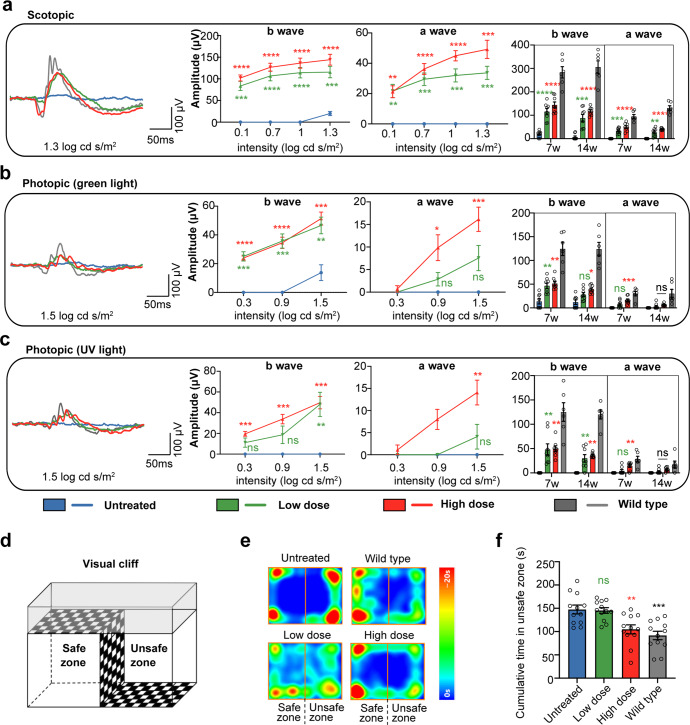
Restoration of retinal and visual function in *rd12* mice after prime editing. Representative ERG waveforms (left), b- and a-wave amplitudes at 5 weeks post injection (7 weeks of age) (middle two), and the comparison of the b- and a-wave amplitudes between 5 weeks and 12 weeks post injection (right) under scotopic (**a**), green light photopic (**b**) and UV light photopic (**c**) conditions. The representative ERG waveforms on the left are recorded under the stimulation at a light stimulus of 1.3 log cd s/m^2^ in scotopic conditions and 1.5 log cd s/m^2^ in photopic conditions. The b- and a-wave amplitude comparisons on the right are under the same conditions. Two-way ANOVA and *post hoc* Dunnett’s for the comparison to untreated group in middle graphs, and own-way ANOVA and *post hoc* Dunnett’s for the comparison to untreated group in right graphs. *P < 0.05, **p < 0.01, ***P < 0.0001, ****P < 0.00001, ns nonsignificant difference. **d** Visual cliff test assembly composed of a safe zone and an unsafe zone. The cumulative time the mice spent in the safe and unsafe zones was recorded. **e** The representative heatmap representing time spent at each position related to the place preference of WT, untreated and treated mice at 5 weeks post injection. **f** The cumulative time spent in the unsafe zone at 5 weeks post injection of WT, untreated and treated mice. Data are shown as mean ± SEM, *n* = 12 for each group. One-way ANOVA and *post hoc* Dunnett’s test were used for comparisons to the untreated group. **p < 0.01, ***p < 0.001; ns nonsignificant difference

The scotopic and photopic ERG showed that eyes treated with dual-AAV8-split-PE at both low and high doses exhibited prominent b- and a-waves at 5 weeks post injection at all stimulus intensities. Untreated eyes showed detectable, but severely abnormal, responses at only the highest stimulus in scotopic and green light photopic ERG (Fig. 5a, b). No response was elicited by UV light at any of the tested light intensities in untreated *rd12* mice (Fig. 5c). In the high-dose group, the rescue was more prominent, with the b- and a-wave amplitudes improved by 43.9% and 51.9%, respectively, at the highest stimulus in scotopic ERG; 30.2% and 53.9% at the highest stimulus in green light photopic ERG; and 39.7% and 49.7% at the highest stimulus in UV light photopic ERG. At 12 weeks after injection, we observed a slight decrease in scotopic and photopic ERG rescue in both the high- and low-dose groups compared to 5 weeks post injection (Fig. 5a–c). Photoreceptor degeneration in the unedited area might be a factor because the ERG response was further decreased in untreated *rd12* mice.

Visual function was tested in wild-type (WT) mice and treated and untreated *rd12* mice using the visual cliff test, which depends on animals’ intrinsic propensity to avoid the deep side of a visual cliff field (Fig. 5d).^18^ The cumulative time the mice spent in the safe and unsafe zones was recorded. The results at 5 weeks post injection showed that untreated *rd12* mice had no preference for the safe or unsafe zone (safe: 147.5 ± 9.4 s; unsafe: 152.5 ± 9.4 s), while WT mice spent less time in the unsafe zone (91.7 ± 9.1 s). Even if ERG showed improved retinal function in both the low- and high-dose groups, only the high-dose group showed significant improvement in visual function, with a decrease in time spent in the unsafe zone compared with untreated mice (104.9 ± 9.7 s vs 152.5 ± 9.4 s, *P* < 0.01) (Fig. 5e, f). The visual cliff test at 12 weeks post injection showed the same tendency (Supplementary Fig. S7).

### Prime editing slowed cone degeneration and rescued cone morphology

**Fig. 6:**
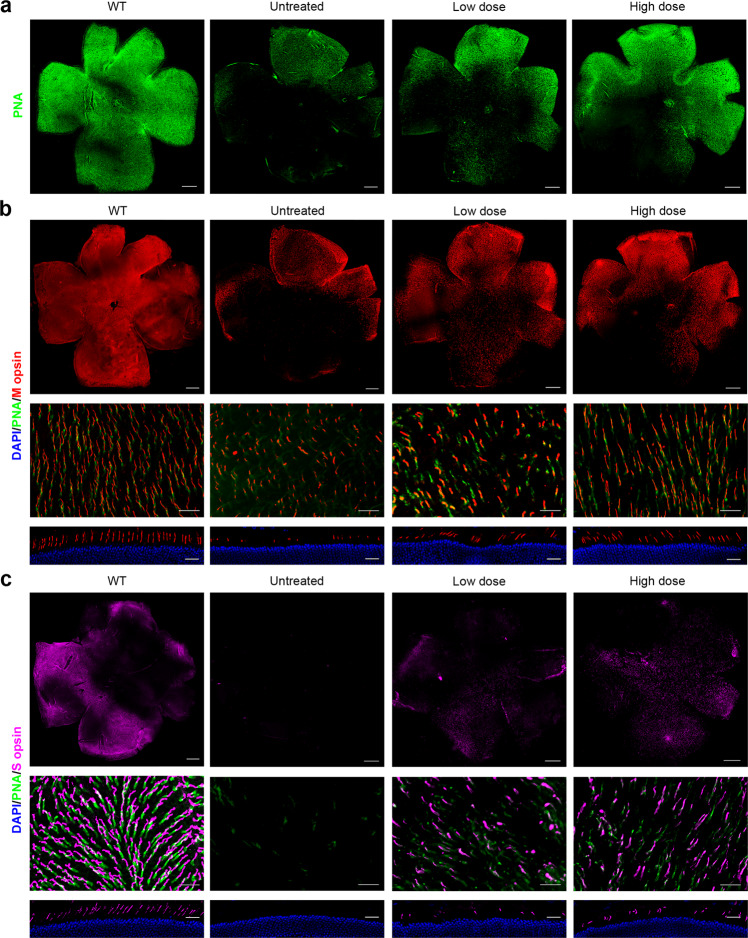
Cone survival in PE3-treated *rd12* eyes. Twelve weeks after injection, eyes were stained with PNA (green) and M- (red) and S-opsin (purple) antibodies. **a** Retinal flatmounts labeled with PNA. Retinal flatmounts at low and high power and cryosections labeled with M-opsin (**b**) or S-opsin (**c**) antibody and PNA. DAPI, blue. Scale bars, 500 μm for flatmounts at low power; 25 μm for flatmounts at high power and eye cross sections

It has been reported that the absence of RPE65 results in rapid cone degeneration.^19^ According to the above photopic ERG results, partial cone function of the treated mice was preserved. To determine the overall cone rescue of prime editing, retinal flatmounts were stained with PNA-lectin, which selectively binds to the cones, at 12 weeks post injection. In untreated *rd12* mice, only a few cones were present in the peripheral dorsal quadrant, which is consistent with a previous study.^19^ Cones in eyes treated with low or high doses were rescued to a greater extent in the high-dose group (Fig. 6a).

We stained retinal flatmounts and retinal cryosections with M-opsin- or S-opsin-specific antibodies to learn more about the rescue of M-cones and S-cones (Fig. 6b, c). In untreated eyes, the remaining cones in the dorsal quadrant are mainly M-cones, with almost all S-cones disappeared (in low and high-magnification images of the retinal flatmounts and images of retinal sections) (Fig. 6b, c). In treated eyes, from the low- and high-magnification images of retinal flatmounts, the area with remaining M- and S-cones and the density of M- and S-cones were improved compared with untreated eyes (Fig. 6b, c). The remaining M- and S-cones in each eye were quantified in eight fields (300 μm × 300 μm each field) at 1 mm from the optic nerve (Supplementary Fig. S8a). The average numbers of M- and S-cones in four fields in the dorsal retina and four fields in the ventral retina were compared among the groups (Supplementary Fig. S8b, c). Compared to the retinas of age-matched WT mice, the remaining M-cones in the dorsal retina were 37.9% in the high-dose group and 34.0% in the low-dose group (vs. 22.2% in the untreated group), and in the ventral retina, they were 21.4% in the high-dose group and 13.0% in the low-dose group (vs. 2.6% in untreated groups). The remaining S-cones in the dorsal retina were 64.9% in the high-dose group and 26.4% in the low-dose group (vs. 0.7% in the untreated group), and those in the ventral retina were 42.6% in the high-dose group and 19.5% in the low-dose group (vs. 5.4% in the untreated groups). Moreover, the M-cone outer segment (OS) was longer and became more similar to that in WT eyes (Fig. 6b, second and third row).

## Discussion

In this study, by optimizing the prime editor based on split intein and dual-AAV vectors, we achieved effective and precise prime editing in the eyes of *rd12* mice and demonstrated a substantial level of vision restoration. We showed that prime editors have clinical potential for correcting mutations causing IRDs and restoring visual function. Our research offers a framework for the preclinical development of a prime-editing therapy for other IRDs caused by different mutations.

Although gene replacement therapies have made significant advancements in the treatment of IRDs, they are only currently available for a tiny proportion of recessive IRD mutations.^1^ For dominant IRDs and recessive IRDs caused by mutations of large genes, gene editing provides alternative therapeutic strategies by targeted knockdown or precise correction of mutations.^20^ PE demonstrated a greater editing efficiency than CRISPR nuclease-mediated precise correction and a wider range of target mutations than BE.^3,21^ The in vivo application of PE could considerably increase the potential to treat IRDs with gene editing therapy.

The current standard delivery system used in humans is AAV due to its high infection efficiency and low immune response. However, the limited packaging size of AAV precludes the delivery of PE. A dual-AAV strategy, where the oversized transgenes are split into two segments and packaged into individual AAV vectors, has been developed to address this challenge. Splitting PE directly by untethering nCas9 from the RT, Liu et al. reported a 1.3% editing efficiency in the liver of *Fah*-mutant mice using dual-AAV delivery.^22^ On the other hand, the split-intein approach is most typically employed to split Cas9, base editor (BE) or PE.^7,10,12^ The choices of split intein and split site are important parameters affecting the reconstitution activities. Npu and Rma intein have been used in ABE and CBE split and could achieve comparable editing to the full-length version at the 574, 675 or 685 sites.^12,23^ However, the split site screening for BE split usually ranged from amino acid (AA) 179 to 740 in the Cas9 domain, which is not suitable for PE split.^23^ PE has a larger gene size than BE and the M-MLV RT domain is fused to the C-terminal of nCas9, while in BE, the TadA-TadA* domain is fused to the N-terminal of nCas9.^3,24^ Zhi et al. evaluated the four splitting sites for PE with Rma intein, including sites 994, 1005, 1024 and 1032, and found that Rma split-1024 was the most effective, achieving an editing efficiency of 1.7% at the *Dnmt1* gene in mouse retina.^25^ PE split at site 714 of Cas9 with strict limitations in promoter and poly A length was delivered by dual-AAV8, resulting in an editing efficiency of 3.1% in mouse liver.^26^ Reducing PE size by removing the RNase H domain of M-MLV RT of the original PE2 conferred flexibility in the choice of split site. The size-reduced PE was split at site 574 or 1153 in two separate experiments, resulting in a precise editing efficiency of 13.5% at the *Pcsk9* gene in mouse liver, or 15.9% at the *Dnmt1* gene and less than 1% at the *Pku* gene in mouse liver, respectively.^27,28^ In our study, we screened the split sites within a larger range, from 859 to 1153 AA of the nCas9 domain, with two types of inteins, and Rma split-1105 showed the best performance in vitro, with the frequency of desired editing being 1.7 times over Rma split-1024 and 4 times over Npu split-1153, which were used in previous studies.^25,27^ Along with other optimizations including the orientations of pegRNA and nicking sgRNA in AAV vector and PBS and RT template in pegRNA, we finally achieved up to a 15.9% (average 11.4%) correction rate in the RPE of *rd12* mice, which is comparable to the correction rate of AAV-mediated BE (average 10.6%)^29^ and is nearly 2 times that induced by PE with *trans-splicing* AAV, mediated by the splicing donor and acceptor present in the two transcripts (average 6.4%),^21^ in the same mouse model.

In this study, dual-AAV8 split-PE3 subsequently restored the expression of RPE65 protein in RPE, improved retinal and visual function, and rescued both M- and S-cones. RPE65 is essential for the recycling of all-*trans*-retinal back into 11-*cis*-retinal, the ligand of rod and cone opsins, which plays an important role in the visual cycle. Continuous RPE65 activity is critical to guarantee that cones acquire adequate visual chromophores to sustain normal cone function.^30^ The visual cycle is hampered by loss-of-function mutations in *RPE65*, which contribute to retinal degeneration. Although the mechanism by which RPE65 deficiency causes early cone degeneration is still unclear,^19^ we demonstrated that restoring RPE65 expression using PE-based gene editing can enhance retinal and visual function, restore cone morphology and slow cone loss, as described in *RPE65* gene replacement therapies.^17,19,31^ A gene replacement therapy, voretigene neparvovec-rzyl (Luxturna), has already been approved by the U.S. Food and Drug Administration (FDA) for treating patients with biallelic *RPE65* mutation-associated LCA. It has improved the vision of patients for about the first three years, but the patients showed a continuation of photoreceptor degeneration in the long term.^32,33^ One of the possible reasons is the inadequate or declining level of RPE65 expression of the transgene.^33^ However, with gene correction, the RPE can express RPE65 in the same manner as the normal RPE and is not dependent on the continuous expression of the transgene, which may offer long-term therapy. However, prime editing may still have certain drawbacks, including off-target effects, even if not detected in this study by the current method.

Despite the fact that we demonstrated prime editing of a specific nonsense mutation in the *Rpe65* gene, we hypothesized that IRDs with other mutations may be fixed by selecting a different pegRNA that accommodated the desired alteration. The optimized delivery method in this study may produce a higher editing efficiency when combined with the newly created PEmax and engineered pegRNA,^5,6^ supporting the therapeutic applicability of prime-editing treatment for various forms of IRDs.

## Materials and methods

### Plasmid construction

pSPgRNA (#47108), pCMV-PE2 (#132775), pU6-Sp-pegRNA-GG-acceptor (#132777), and pU6-Sp-pegRNA_HEK3_CTT_ins (#132778) were obtained from Addgene (Watertown, MA). The U6-pegRNA vectors were constructed with pU6-Sp-pegRNA-GG-acceptor and the U6-nicksing sgRNA vectors were constructed with pSPgRNA. pegRNAs were constructed with pU6-Sp-pegRNA-GG-acceptor, as previously reported.^3^ Nicking sgRNAs were generated by T4 ligation of annealed oligos into the BbsI-digested pSPgRNA plasmid. The pegRNA and nicking sgRNA sequences for different targets in HEK293 cells and HEK293-*rd12* cells are listed in Supplementary Tables S1 and S2. By in-fusion cloning of PCR amplicons to restriction enzyme-digested backbones, the N- and C-terminals of PE2 were produced. Every plasmid was confirmed by sequencing.

### Cell line generation

The HEK293-*rd12* mutant cell line was created using CRISPR/Cas9 vectors that were constructed using the pX330 plasmid. The CRISPR/Cas9 plasmid, together with a donor plasmid containing the *Rpe65* mutant gene and a puromycin resistance cassette, was transfected into HEK293 cells. Puromycin was used to select the cells 72 h after transfection, and the monoclonal cells were transferred to 96-well plates and then amplified. After that, cell genomic DNA was extracted for PCR and sequencing validation. Finally, by stably integrating the Rpe65 mutation sequence into the AAVS1 locus, HEK293-*rd12* mutant cell lines were created.

### Cell culture and transfection

DMEM supplemented (Gibco) with 10% fetal bovine serum (FBS) (PAN Biotech) was used to cultivate HEK293 and HEK293-*rd12* cells at 37 °C with 5% CO_2_. Cells were seeded into 24-well plates at 1 × 10^5^ cells per well 24 hours prior to transfection. Full-length PE plasmid, pegRNA plasmid and nicking sgRNA plasmid were transfected at 750 ng, 180 ng, and 250 ng, respectively. Split PE plasmids were transfected at equal molar concentrations. The cells were harvested for western blot or genome sequencing three days after transfection.

### Prime-editing analysis with Sanger sequencing

Sanger sequencing was used to evaluate on-target genomic regions. Base substitutions were further quantified with EditR (http://baseeditr.com)^34^ and small indels were further quantified with TIDE (https://tide.nki.nl).^35^

### Western blot

Cells were lysed in 50 μl ice-cold RIPA buffer (Beyotime) with protease inhibitors (Sigma) by maintaining steady agitation for 45 min at 4 °C to preduce the protein from cell lysates. The RPE-choroid-sclera complex was added to 50 μl RIPA buffer with protease inhibitors before homogenization using a motor tissue grinder (Omni International Corp.), and then maintained with constant agitation for 45 min at 4 °C to prepare the protein from the posterior eye of the mouse. Then, the lysates or homogenous eye cups were centrifuged for 15 min at 13,300 × *g* and 4 °C. A BCA protein assay (Thermo Scientific) was used to quantify the total protein concentration. SDS-PAGE was used to seperate samples (20 μg of the cell lysate and 10 μg of the RPE tissue), which were then transferred to PVDF membranes. The membranes were incubated with primary antibodies at 4 °C overnight after being blocked with 5% non-fat milk in TBST at RT for 2 h. Mouse anti-Cas9 monoclonal antibody (1:1000; Abcam), rabbit anti-RPE65 monoclonal antibody (1:1000; Abcam), and rabbit anti-GAPDH polyclonal antibody (ABclonal) were the primary antibodies used in this study. After washing in TBST, the membranes were incubated with HRP-conjugated secondary antibody at RT for 2 h. The secondary antibodies included goat anti-rabbit IgG (1:10,000; Zsbio) and goat anti-mouse IgG (1:10,000; Zsbio). Blots were imaged by iBrightTM CL1000 imaging systems (Thermo Scientific).

### AAV vector production

All AAV8 vectors were produced by triple plasmid transfection of HEK293 cells as previously described.^36^ Briefly, 10-layer cell stacks (Corning, Corning, NY) were seeded with 3 × 10^8^ HEK293 cells. PEI-based transfections were carried out when the cell confluence reached 75–90%. Plasmids at a ratio of 2:1:1 were used. The PEI-Max/DNA ratio was maintained at 2:1(w/w). For each cell stack, the plasmids and PEI-Max were mixed and vortexed and then incubated for 15 min at RT before being added to 1 liter of serum-free DMEM (SFM). The PEI-Max/DNA mixture was added to the stack in place of the culture medium, and it was then incubated at 37 °C with 5% CO_2_. Then, 500 ml of SFM was added at 72 h after transfection, and incubation was carried out for an additional 48 h. Then, the virus was harvested and purified by iodixanol gradient ultracentrifugation. The digital droplet polymerase chain reaction (ddPCR) was used to measure the AAV8 vector’s genome titer (GC/ml), with forward primer 5′-GCAGACATGATAAGATACATTGATGAGTT-3′, reverse primer 5′-AGCAATAGCATCACAAATTTCACAA-3′, and probe 5′-Fam-AGCATTTTTTTCACTGCATTCTAGTTGTGGTTTGTC-BHQ-3′. Utilizing the end-point chromogenic endotoxin test kit (Xiamen Bioendo Technology Co.,Ltd, Xiamen, China), all vectors used in this study passed the endotoxin assay.

### Animals

*rd12* mice were obtained from the Jackson Laboratory (Bar Harbor). WT C57BL/6J mice were obtained from Chengdu Dossy Experimental Animals Co., Ltd. (Chengdu, China). Animals were maintained in a 12 h cycle of light and darkness, with unrestricted access to food and water. In this investigation, mice were anesthetized intraperitoneally with xylazine (12 mg/kg) and ketamine (80 mg/kg) unless otherwise stated. The pupils were dilated with an eye drop that inlcuded 0.5% tropicamide and 0.5% phenylephrine hydrochloride. The Institutional Animal Care and Concern Committee of Sichuan University gave its approval for the animal experiments, and the committee’s rules were followed when caring for the animals.

### Subretinal injection

At the age of two weeks, subretinal injections were performed on mice. Under a stereomicroscope, a 31-gauge needle was used to create a limbal hole after isoflurane inhalation anesthesia and pupil dilatation. The subretinal space was targeted by a blunt 33-gauge needle (Hamilton Co.) inserted through the hole without lens damage. Each eye was injected with 1 μl of AAV8 (the ratio of AAV8.nksgRNA.N-PE2.pegRNA and AAV8.N-PE2 is 1:1). Immediately after injection, the fundus was observed by fundus imaging microscopy (Micron IV, Phoenix Research Labs). Successful injections were those that created subretinal blebs but no significant vitreous hemorrhage or subretinal bleeding. Following fundus observation, the cornea was treated with ofloxacin eye ointment.

### Immunohistochemistry of eye cryosections

Mice were euthanized 12 weeks after injection. For frozen cross sections, eyes were removed, fixed in 4% paraformaldehyde (PFA)/PBS at RT for 1 hr, and then washed three times in PBS for 5 min each. The center of the cornea was cut off before cryoprotection in 30% sucrose/PBS overnight at 4 °C. Then the eye cups were embedded in O.C.T. (Sakura Finetek USA, Inc.). After rinsing with PBS for 5 min, frozen retinal slices with a thickness of 10 μm were blocked with 5% normal goat serum and 0.2% Triton X-100 in PBS for 1 hr at RT. The slices were then incubated with primary antibodies in blocking solution overnight at 4 °C. Rabbit anti-RPE65 (1:250 dilution; Abcam), rabbit anti-S opsin (1:50 dilution; Novus Biologicals), and rabbit anti-M opsin (1:1000 dilution; Novus Biologicals) were the primary antibodies used in this study. After washing 3 times in PBST for 10 min each, slices were treated with goat anti-rabbit IgG Alexa Fluor 594 (1:1000 dilution; Abcam) for 1 hr at RT. After 3 washes in PBS for 10 min each, slices were stained with DAPI and photographed using a confocal laser microscope (Nikon, Tokyo, Japan).

### Immunohistochemistry of RPE and retinal flatmouts and quantification

After enucleation, the eyes were first marked on the 12 o’clock position of the limbus, and the cornea, lens, vitreous and optic nerve bud were gently removed. After carefully separating the whole retina from the RPE/choroid/sclera complex, four radial cuts were performed toward the optic nerve head to flatten the retina and RPE/choroid/sclera. The RPE/choroid/sclera or retina was fixed in 4% paraformaldehyde in 0.1 M PBS for 1 h at RT before being rinsed three times for 10 min each time. The samples were then blocked with 10% normal goat serum for 1 h at RT before being stained with primary antibody for 48 hr at 4 °C. The primary antibodies for RPE/choroid/sclera included rabbit anti-RPE65 (1:250; Abcam) and rat anti-ZO-1 (1:100; Santa Cruz Biotechnology); those for retina included FITC-conjugated peanut agglutinin (1:200; Sigma), rabbit anti-S opsin (1:50 dilution; Novus Biologicals), and rabbit anti-M opsin (1:1000 dilution; Novus Biologicals). Following three 15-min PBST washes, the specimens were incubated with secondary antibodies for 1 h at RT. Goat anti-rabbit IgG Alexa Fluor 594 (1:1000; Abcam) and goat anti-rat IgG Alexa Fluor 488 (1:1000; Abcam) were the secondary antibodies employed in this study. The specimens were then photographed using a confocal laser microscope (Nikon) after being washed in PBS three times for 15 min each. To count the number of M- and S-cones in a retinal flatmount, images of eight fields (300 μm × 300 μm) 1 mm away from the optic nerve were taken. Using ImageJ (Image Processing and Analysis in Java; the National Institutes of Health), quantification in each field was carried out. Prior to photography, the RPE flatmounts were stained with DAPI. ImageJ was used to quantify the RPE65^+^ area.

### Deep sequencing and data analysis

Mouse RPE tissue was isolated by the mechanical dissociation method.^37^ Briefly, the posterior eye cup (sclera-choroid-RPE) was flash frozen with liquid nitrogen and then PBS (200 μl) was used to flush onto the RPE side. The freeze-thaw PBS flushing was repeated more than 40 times. Then, genomic DNA of the RPE was extracted using a QIAamp DNA Micro Kit (Qiagen). Subsequent nested PCR was used to generate amplicons (200 to 300 bp) of on-target and predicted off-target sites for *Rpe65*. The primers used for amplifying on-target and off-target sites are listed in Supplementary Table S3. The top 8 potential off-target sites for pegRNA and top 5 potential off-target sites for nicking sgRNA were identified using CRISPOR.^14^ The primers that included barcode sequences were used in the second round of PCR. After gel purification with the E.Z.N.A. Gel Extraction Kit (Omega), the amplicons were sequenced using the NovaSeq (Illumina) platform (2 × 150 bp paired end, Personal Biotechnology Co., Ltd, Shanghai, China). Substitution and indel frequencies were quantified as the percentage of total sequencing reads. To rule out errors caused by PCR amplification and sequencing, untreated mice were utilized as negative controls. To quantify the desired editing of prime editing, amplicon sequences were aligned to reference sequences using CISPResso2.^38^ Substitutions and indels of the on-target and off-target sites were analyzed using integrated digital error suppression (iDES) algorithm.^39^ The SNP was measured over a 40-bp range centered on the pegRNA or nicking sgRNA cut site, and the indel was measured over a 100-bp range centered on the pegRNA or nicking sgRNA cut site.

### ERG

Scotopic and photopic ERG was used to evaluate the retinal function of mice. ERG was recorded according to the manufacturer’s instructions for the Phoenix Ganzfeld ERG (Phoenix Research Labs). Mice were dark adapted for 16 hr, after which all preparations were carried out in dim red light. A heating pad was used to maintain the body temperature of the mice after anesthesia. The ground electrode was positioned subcutaneously in the tail, while the reference electrode was positioned subcutaneously on the forehead between the ears. After pupil dilation, a corneal electrode was placed on the cornea with 2.5% hypromellose. Scotopic ERG was recorded at four stimulus intensities varying between 0.1 and 1.3 log cd s/m^2^. For photopic ERG, the mouse eye was exposed to constant green and UV light (1.3 log cd/m^2^) for 5 minutes. Two series of recordings were then performed with green light flashes (504 nm, from 0.3 to 1.5 log cd s/m^2^) and UV light flashes (365 nm, from 0.3 to 1.5 log cd s/m^2^) to selectively excite the M-cone and S-cone pathways.

### Visual cliff test

A visual cliff test was used to assess visual function as previously described,^40,41^ with slight modifications. The apparatus consisted of two plastic boxes. Half of the top of the first box (length: 50 cm; width: 40 cm; height: 50 cm) is covered, while the other half is open. On the top of the covered half, a checkerboard pattern with black and white squares (2 cm × 2 cm) was settled, which continued to the bottom of the opened half. The second box had an open side facing upward, a transparent bottom, and black walls around it. The second box was placed on the top of the first box (Fig. 5d). In this setting, the second box represented an open field composed of two equal sections: the section with the checkboard pattern on the top of the first box (length: 45 cm; width: 20 cm), called the “safe zone” (or “shallow end”), and the section with a cliff between the transparent bottom of the top box and the checkerboard-patterned bottom, called the “unsafe zone” (or “deep end”). Homogenous luminance in the apparatus was set at 10 lux at the height of the mouse eyes.

During the test, a mouse was left free to roam the entire open field for 300 s after being set up in the corner of the shallow end section. Its behavior was recorded by a video camera, and the time the test animals spent in the safe or unsafe zone was analyzed by ANY maze software (Global Biotech Inc., Shanghai, China). Between each mouse, the device was cleaned with water and 70% ethanol. All analyses were performed by a single operator who was blinded to mouse genotypes and treatment.

### Statistical analysis

The statistical tests used for each experiment are stated in the corresponding figure legends. Statistical analysis was performed using GraphPad Prism (University of California, San Diego, California).

## Supplementary information

SUPPLEMENTAL MATERIAL

## Data Availability

All data are presented in the manuscript or the supplementary materials. The deep-sequencing data of this study have been deposited in the Sequence Read Archive under accession number PRJNA864607.
