# Supplementary material for: Dual-AAV split prime editor corrects the mutation and phenotype in mice with inherited retinal degeneration
Source: Signal Transduct Target Ther. 2023 Feb 6;8:57. doi: 10.1038/s41392-022-01234-1 (PMC9899767; doi:10.1038/s41392-022-01234-1)
Supplement: Supplementary file 1 — SUPPLEMENTAL MATERIAL [file 41392_2022_1234_MOESM1_ESM.docx]

Supplementary Materials for

Dual-AAV split prime editor corrects the mutation and phenotype in mice with inherited retinal degeneration

**Authors:** Kaiqin She^1^, Yi Liu^2^, Qinyu Zhao^2^, Xiu Jin^2^, Yiliu Yang^1^, Jing Su^2^, Ruiting Li^2^, Li Song^2^, Jianlu Xiao^2^, Shaohua Yao^2^, Fang Lu*^1^, Yuquan Wei^2^, Yang Yang*^1,2^ **Affliations**: ^1^Department of Ophthalmology, West China Hospital, Sichuan University, Chengdu, Sichuan, China. ^2^State Key Laboratory of Biotherapy and Cancer Center, West China Hospital, Sichuan University and Collaborative Innovation Center, Chengdu, Sichuan, China.

Correspondence to: Fang Lu ([lufang@wchscu.cn](mailto:lufang@wchscu.cn)); Yang Yang ([yang2012@scu.edu.cn](mailto:yang2012@scu.edu.cn))

**This PDF file includes:**

Materials and Methods

Figures. S1 to S8

Tables S1 to S3

Materials and Methods

Estimating the transfected area of RPE

The 2-week-old *rd12* mice were injected with 3×10^9^ or 1×10^10^ GC/eye dual-AAV PE3 system along with 1×10^8^ GC/eye AAV8-CMV-EGFP. After one week, the fundus was photographed by retinal imaging microscopy (Micron IV, Phoenix Research Labs). Twelve weeks after the injection, RPE flatmounts were prepared. Briefly, after enucleation, the cornea, lens, vitreous, retina, and optic nerve bud were carefully removed to generate posterior eye cups containing the RPE/choroid/sclera. Then four radial cuts were made toward the optic nerve head to flatten the RPE/choroid/sclera. The RPE/choroid/sclera or retina was fixed in 4% paraformaldehyde in 0.1 M PBS for 1 hr at RT and then washed in PBS 3 times for 10 min. Then the RPE flatmounts were imaged with a confocal laser microscope (Nikon). To estimate the transfected area of the RPE, the margin of the GFP+ area was outlined, and the percentage of the transfected area was calculated using ImageJ (Image Processing and Analysis in Java; the National Institutes of Health).


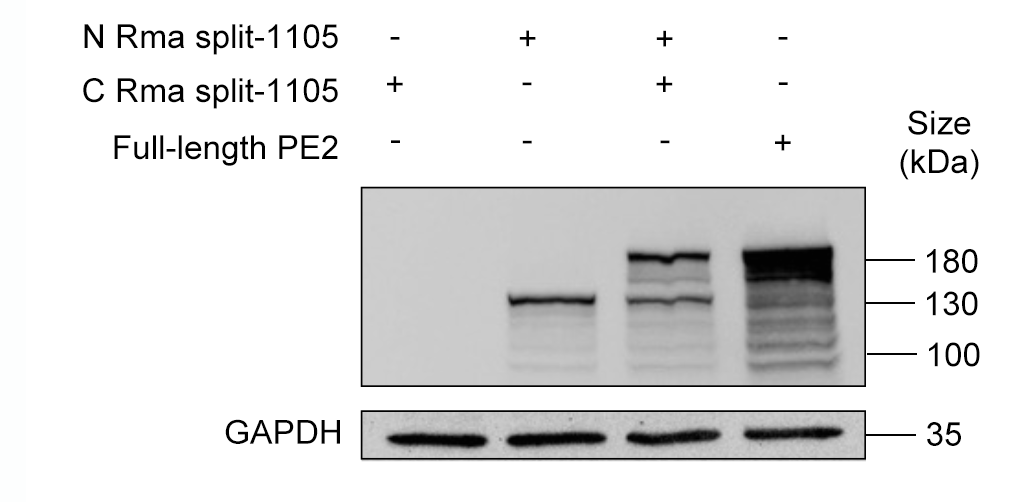


Figure. S1.

Western blot analysis to detect full-length PE2 expression in cells co-transfected with the N terminal and C terminal of PE2 split by Rma-1105. GAPDH (36 kDa) was used as a loading control.

**
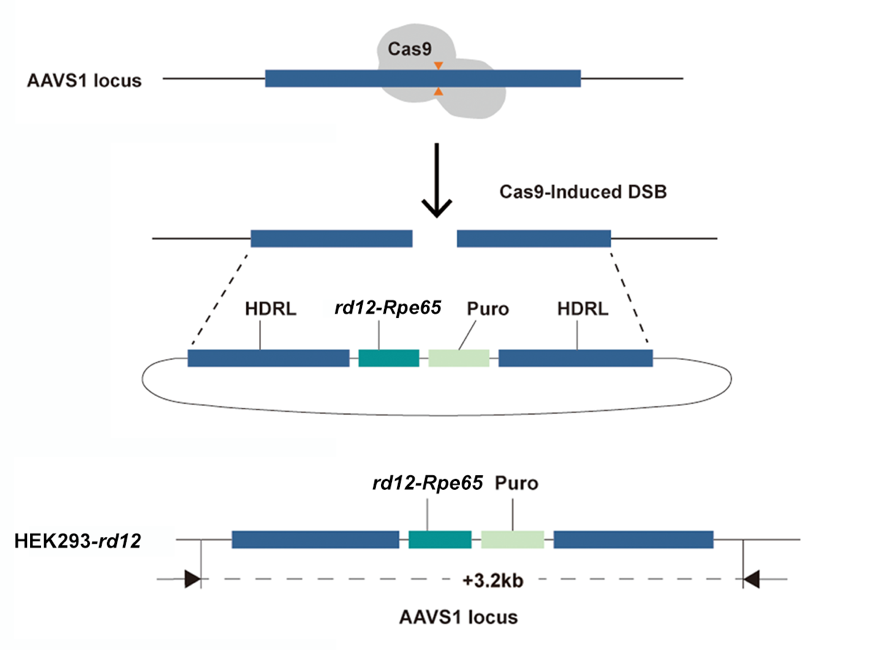
**

Figure. S2.

Schematic of the generation of the HEK293-rd12 mutant cell line by stably integrating the rd12 mutant Rpe65 sequence into the AAVS1 genomic locus using CRISPR/Cas9.


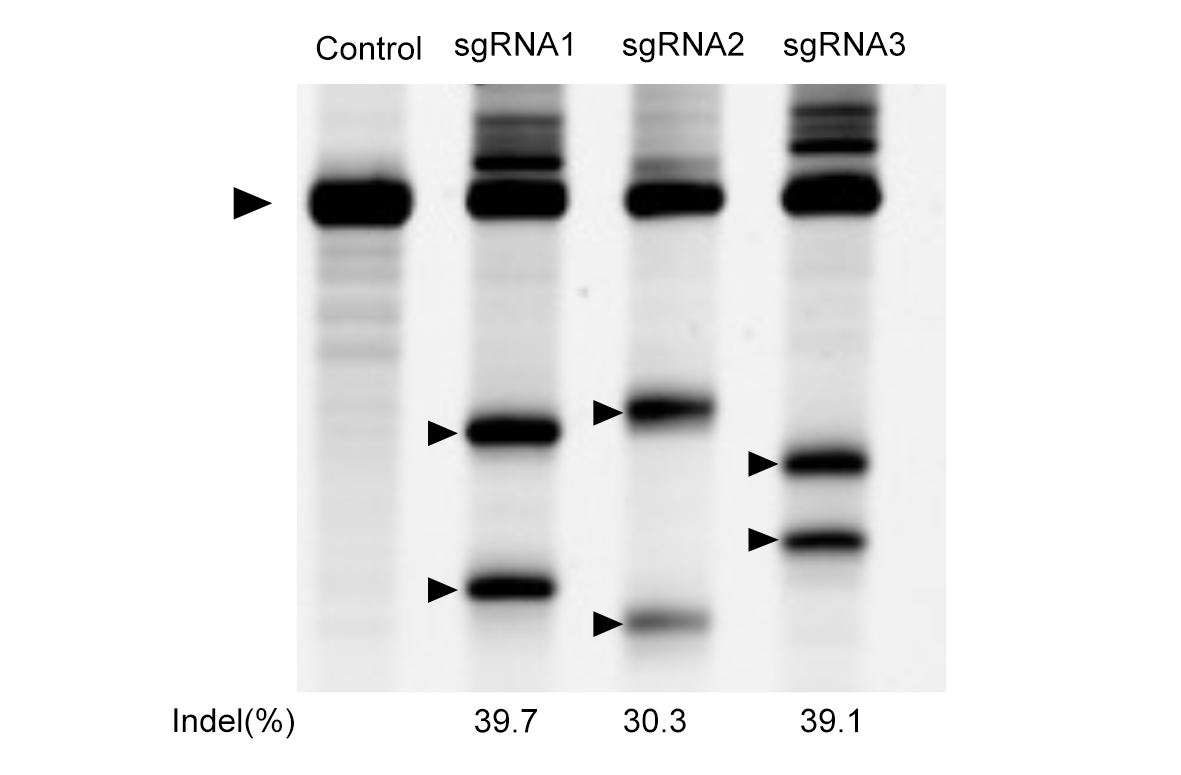


Figure. S3.

Screening of the three spacers of pegRNA targeting *Rpe65* mutation by surveyor nuclease assay.


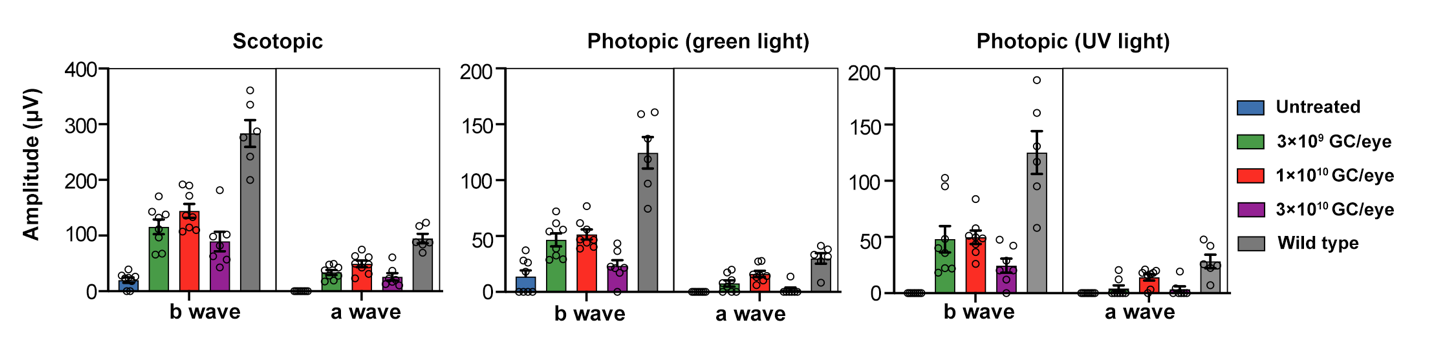


Figure. S4.

Comparison of the b- and a-wave amplitudes of scotopic (left), green light photopic (middle) and UV light photopic (right) among different dose groups at 5 weeks post injection.


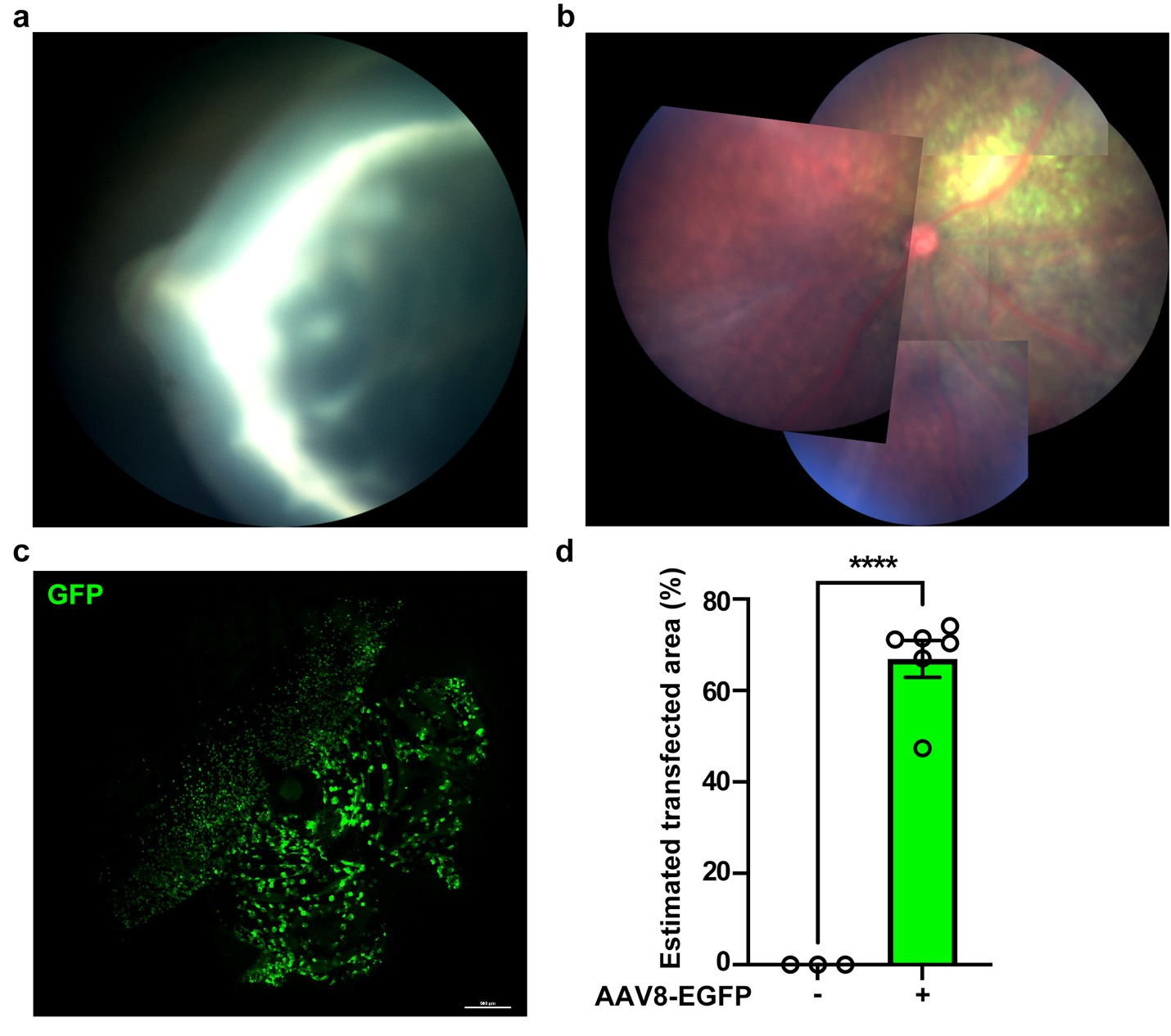


Figure. S5.

Estimating the transfected area of the RPE by injection with 1×10^8^ GC/eye AAV8-EGFP along with the dual-AAV PE3. **a.** The injection bleb. **b.** The montage of the fundus photographs taken one week after the injection. The transfected area was green. Twelve weeks after the injection, RPE flatmounts were prepared to estimate the transfected area **(c&d)**. **c.** Representative RPE flatmount. **d.** Estimated transfected area from the low magnification images (n = 3 in the group without AAV8-EGFP; n=6 in the group with AAV8-EGFP) of the RPE flatmounts based on immunofluorescence. Means ± SEM are shown. Student’s *t* test. ****p<0.0001.


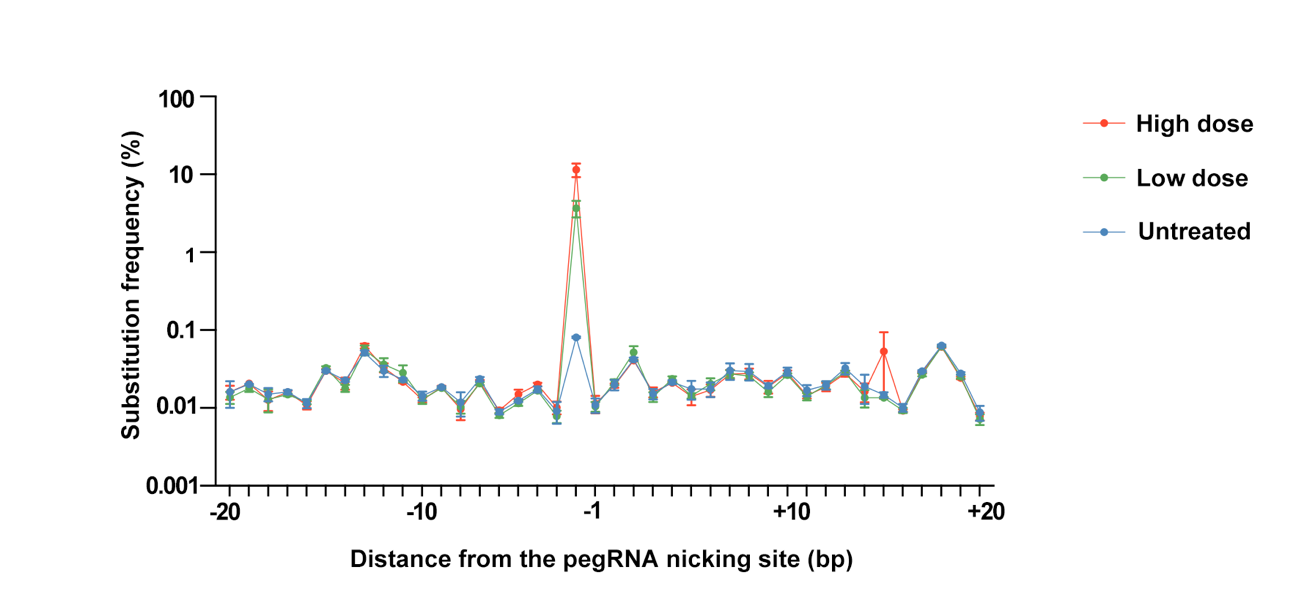


Figure. S6.

Substitution frequencies at positions ranging from −20 bp to +20 bp of the pegRNA nicking site in PE3-treated mice. The targeted position is at- 2. Data are mean ± SEM, n = 3 for each group.


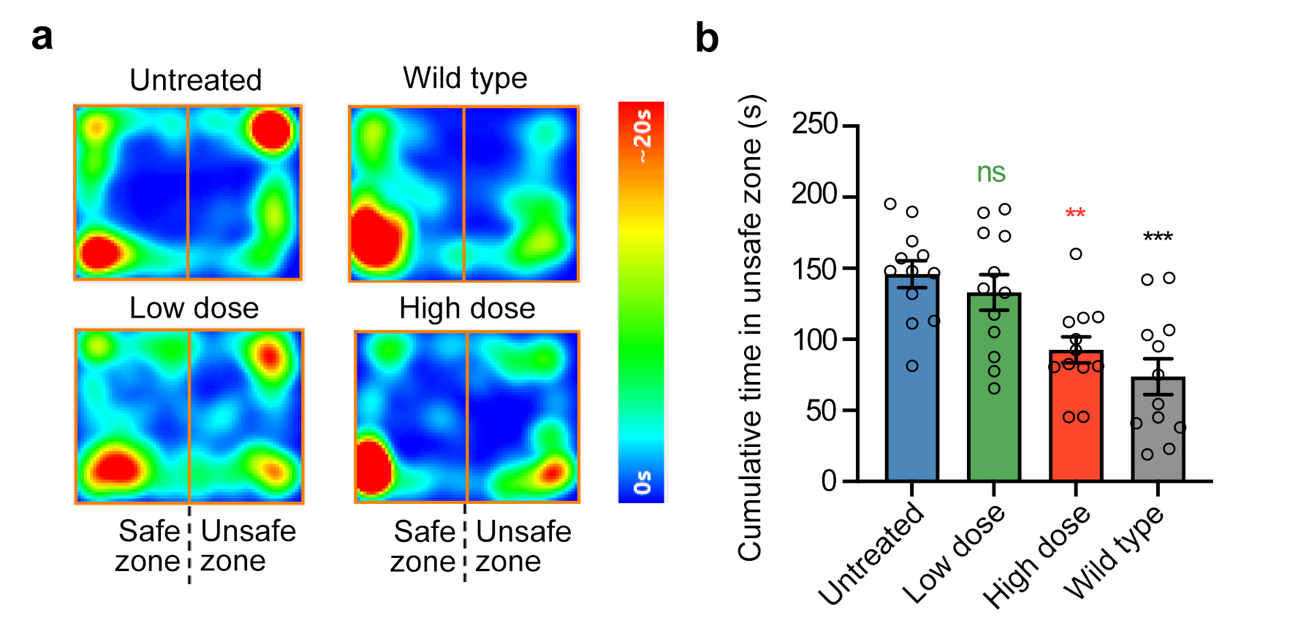


Figure. S7.

Cliff test of the PE-treated mice at 12 weeks post injection. **a.** The representative heatmap representing time spent at each position related to the place preference of WT, untreated and treated mice. **f.** The cumulative time spent in the unsafe zone at 12 weeks post injection of WT, untreated and treated mice. Data are shown as mean ± SEM, n=12 for each group. One-way ANOVA and *post hoc* Dunnett’s test were used for comparisons to the untreated group. **p < 0.01, ***p < 0.001; ns. nonsignificant difference.


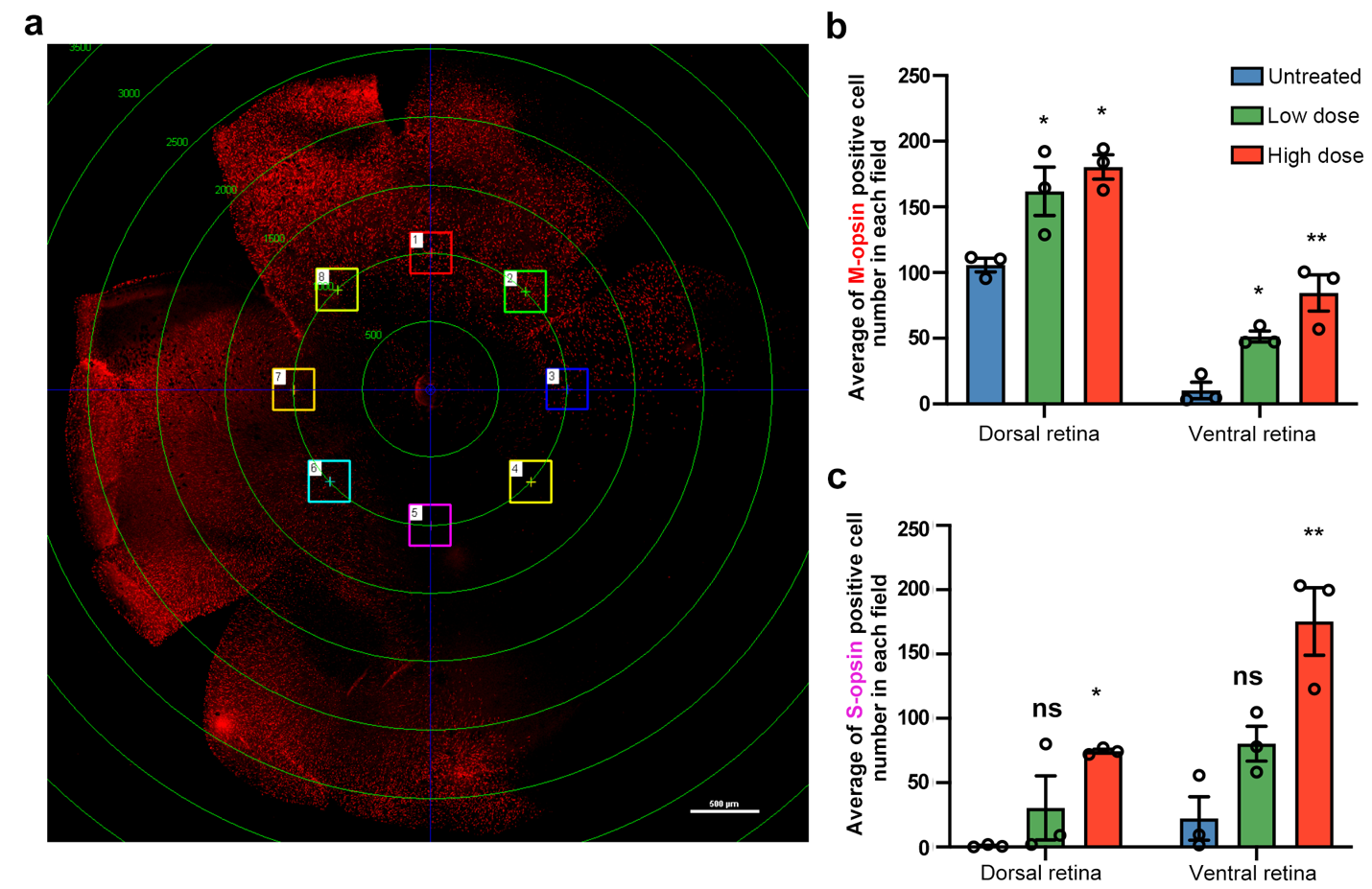
Figure. S8.

Quantification of M- and S-opsin-positive cones. **a.** A representative image of eight fields (300 μm×300 μm each field) at 1 mm from the optic nerve. **b.** Quantification of the averagenumber of M-cones in the dorsal and ventral retina. Data are shown as mean ± SEM, n=3 for each group. One-way ANOVA and *post hoc* Dunnett’s test were used for comparisons to the untreated group. *p < 0.05, **p < 0.01. **c.** Quantification of the average number of S-cones in the dorsal and ventral retina. Data are shown as mean ± SEM, n=3 for each group. One-way ANOVA and *post hoc* Dunnett’s test were used for comparisons to the untreated group. *p < 0.05, **p < 0.01; ns. nonsignificant difference.

Table S1.

Sequence of pegRNAs and nicking sgRNAs used in HEK293 cells.

| **pegRNA** | **spacer sequnce** | **3’ extension sequnce** | **PBS length (nt)** | **RT template length (nt)** |
| --- | --- | --- | --- | --- |
| pegHEK3_1CTTins | GGCCCAGACTGAGCACGTGA | TCTGCCATCAAAGCGTGCTCAGTCTG | 13 | 10 |
| pegRNF2_6GtoA | GTCATCTTAGTCATTACCTG | AACGAACATCTCAGGTAATGACTAAGATG | 15 | 14 |
| pegVEGFA_2-4GAGdel | GATGTCTGCAGGCCAGATGA | AATGTGCCATCTGGAGCCATCTGGCCTGCAGA | 13 | 19 |
| **nicking sgRNA** | **spacer sequnce** |  |  |  |
| HEK3_+90 | GTCAACCAGTATCCCGGTGC |  |  |  |
| RNF2_+41 | GTCAACCATTAAGCAAAACAT |  |  |  |
| VEGFA_+57 | GATGTACAGAGAGCCCAGGGC |  |  |  |

Table S2.

Sequence of pegRNAs and nicking sgRNAs used in HEK293-*rd12* cells.

| **pegRNA** | **spacer** | **3’ extension sequnce** | **PBS length (nt)** | **RT template length (nt)** |
| --- | --- | --- | --- | --- |
| pegRNA-Rpe65-1 | AGAGCCCTGGCCCACATCAG | TCCTCCGATGTGGGC | 8 | 7 |
| pegRNA-Rpe65-2 | AGAGCCCTGGCCCACATCAG | TCCTCCGATGTGGGCCA | 10 | 7 |
| pegRNA-Rpe65-3 | AGAGCCCTGGCCCACATCAG | TCCTCCGATGTGGGCCAGG | 12 | 7 |
| pegRNA-Rpe65-4 | AGAGCCCTGGCCCACATCAG | TCCTCCGATGTGGGCCAGGGC | 14 | 7 |
| pegRNA-Rpe65-5 | AGAGCCCTGGCCCACATCAG | TCCTCCGATGTGGGCCAGGGCTC | 16 | 7 |
| pegRNA-Rpe65-6 | AGAGCCCTGGCCCACATCAG | TCTCCTCCGATGTGGGC | 8 | 9 |
| pegRNA-Rpe65-7 | AGAGCCCTGGCCCACATCAG | TCTCCTCCGATGTGGGCCA | 10 | 9 |
| pegRNA-Rpe65-8 | AGAGCCCTGGCCCACATCAG | TCTCCTCCGATGTGGGCCAGG | 12 | 9 |
| pegRNA-Rpe65-9 | AGAGCCCTGGCCCACATCAG | TCTCCTCCGATGTGGGCCAGGGC | 14 | 9 |
| pegRNA-Rpe65-10 | AGAGCCCTGGCCCACATCAG | TCTCCTCCGATGTGGGCCAGGGCTC | 16 | 9 |
| pegRNA-Rpe65-11 | AGAGCCCTGGCCCACATCAG | AGTCTCCTCCGATGTGGGC | 8 | 11 |
| pegRNA-Rpe65-12 | AGAGCCCTGGCCCACATCAG | AGTCTCCTCCGATGTGGGCCA | 10 | 11 |
| pegRNA-Rpe65-13 | AGAGCCCTGGCCCACATCAG | AGTCTCCTCCGATGTGGGCCAGG | 12 | 11 |
| pegRNA-Rpe65-14 | AGAGCCCTGGCCCACATCAG | AGTCTCCTCCGATGTGGGCCAGGGC | 14 | 11 |
| pegRNA-Rpe65-15 | AGAGCCCTGGCCCACATCAG | AGTCTCCTCCGATGTGGGCCAGGGCTC | 16 | 11 |
| pegRNA-Rpe65-16 | AGAGCCCTGGCCCACATCAG | GCAGTCTCCTCCGATGTGGGC | 8 | 13 |
| pegRNA-Rpe65-17 | AGAGCCCTGGCCCACATCAG | GCAGTCTCCTCCGATGTGGGCCA | 10 | 13 |
| pegRNA-Rpe65-18 | AGAGCCCTGGCCCACATCAG | GCAGTCTCCTCCGATGTGGGCCAGG | 12 | 13 |
| pegRNA-Rpe65-19 | AGAGCCCTGGCCCACATCAG | GCAGTCTCCTCCGATGTGGGCCAGGGC | 14 | 13 |
| pegRNA-Rpe65-20 | AGAGCCCTGGCCCACATCAG | GCAGTCTCCTCCGATGTGGGCCAGGGCTC | 16 | 13 |
| pegRNA-Rpe65-21 | AGAGCCCTGGCCCACATCAG | TGGCAGTCTCCTCCGATGTGGGC | 8 | 15 |
| pegRNA-Rpe65-22 | AGAGCCCTGGCCCACATCAG | TGGCAGTCTCCTCCGATGTGGGCCA | 10 | 15 |
| pegRNA-Rpe65-23 | AGAGCCCTGGCCCACATCAG | TGGCAGTCTCCTCCGATGTGGGCCAGG | 12 | 15 |
| pegRNA-Rpe65-24 | AGAGCCCTGGCCCACATCAG | TGGCAGTCTCCTCCGATGTGGGCCAGGGC | 14 | 15 |
| pegRNA-Rpe65-25 | AGAGCCCTGGCCCACATCAG | TGGCAGTCTCCTCCGATGTGGGCCAGGGCTC | 16 | 15 |
| **nicking sgRNA** | **spacer sequnce** |  |  |  |
| Rpe65_-109 | GCCATGTCACATACCACAGA |  |  |  |
| Rpe65_-51 | CCTTTCTATCACCTGTTTGA |  |  |  |
| Rpe65_-7 | GTCTCCTCCGATGTGGGCCA |  |  |  |
| Rpe65_+42 | CTGATATCTCACTTTGCTGC |  |  |  |
| Rpe65_+94 | TAAGCTGACAAATAACAAAT |  |  |  |

Table S3.

PCR primer sequences for detecting on-target and potential off-target effects by deep sequencing

| **Primer Name** | **Sequence** | **Primer Name** | **Sequence** |
| --- | --- | --- | --- |
| ON_P1Fwd | CAGCCAACATTCTTGCTGCT | nOT1_P1Fwd | CCTATCAAAGAACTCCCTCC |
| ON_P1Rev | ATTCCCTACCAGATGCCATC | nOT1_P1Rev | GTTCTCAGCAATACATCCTC |
| ON_P2Fwd | ACCACCTGATATCTCACTTTGCT | nOT1_P2Fwd | CACTTCCTGCCTTGGCCATA |
| ON_P2Rev | TGGACTTACCTTCTGTGGTA | nOT1_P2Rev | ACTGCAGACCACATAACCCG |
| OT1_P1Fwd | GACAGCAGGGAAGAGCAACA | nOT2_P1Fwd | GGTTAATTCCCAGGTATGGG |
| OT1_P1Rev | TGTGTGAGATGAGCTGGAGT | nOT2_P1Rev | AGGCAACATGAATACTGAGC |
| OT1_P2Fwd | GAAAGGCTCAGAGAACTGCCT | nOT2_P2Fwd | CTGTGGTTCCAGTGACTTCT |
| OT1_P2Rev | ACGTTGGTCTGGAATCTTGGT | nOT2_P2Rev | CCCTGAAGTTCAGGTTCTAT |
| OT2_P1Fwd | ATAGAGACAAGCAGGCACAC | nOT3_P1Fwd | CCCCACAGACTCATGTCACC |
| OT2_P1Rev | AGCCACAGCCACCATAGAGA | nOT3_P1Rev | CTGGCCCCACAATGGATTCT |
| OT2_P2Fwd | CCCAGGTGATCAAAGGCCAT | nOT3_P2Fwd | CAGACAATAGAAGCTCAGCT |
| OT2_P2Rev | ATAATTGTGGCGCAGGAGGG | nOT3_P2Rev | GCTGAGCTTACAGTTCTGTG |
| OT3_P1Fwd | CAGCAGTTCCTCGGTCCACA | nOT4_P1Fwd | TGCATTCCTTTGGAAGCCAG |
| OT3_P1Rev | CCTGTGACAATCTGATGGAG | nOT4_P1Rev | CCCGTGGTGTCTTAATATGT |
| OT3_P2Fwd | TGAGAGCAAGCAAGCAAAGA | nOT4_P2Fwd | CACAGCCTTGAGCTGAATGA |
| OT3_P2Rev | TGAAGAGCACACCTGTGAGG | nOT4_P2Rev | CTTTGGAGATTTGCTATGCG |
| OT4_P1Fwd | TGTGTAACCTTGGTGTGCTG | nOT5_P1Fwd | CCACATGCTTGGTGATGTCC |
| OT4_P1Rev | ACAGGCAAAGGGAACTGGCC | nOT5_P1Rev | ATCAGACAGAATAGGAGGGC |
| OT4_P2Fwd | GGAGTTAACCTGATGACCTG | nOT5_P2Fwd | TTGGCTCACAGTTTGAGGAT |
| OT4_P2Rev | ACCAGCAAAGCAGTGAGATT | nOT5_P2Rev | GGTCCAACAGAATAGTAACAC |
| OT5_P1Fwd | TCTGCAGTTCCTCAAGGCAG |  |  |
| OT5_P1Rev | CCTGGGAGGATCAGAGGTCA |  |  |
| OT5_P2Fwd | TCAGTGCTTGTCGGTTCCAA |  |  |
| OT5_P2Rev | GAGGCCAGCCTACTCTAAAC |  |  |
| OT6_P1Fwd | TGTGCGAAGAGTACAGCAGG |  |  |
| OT6_P1Rev | GAACGTACTGGGCCAATCCA |  |  |
| OT6_P2Fwd | TCGCCATCAGATGTCCACTG |  |  |
| OT6_P2Rev | CAGCACCTGCTGGATCTGCA |  |  |
| OT7_P1Fwd | GAAAGTCATAGCCCCACCCC |  |  |
| OT7_P1Rev | TTCGTGGGTCTGAAAGGCTC |  |  |
| OT7_P2Fwd | TTCGCTGGGAGATGTTCGAG |  |  |
| OT7_P2Rev | CAGAGTAGGTTGGTGGGCAG |  |  |
| OT8_P1Fwd | AGGATGCTGGTCTGATATGGA |  |  |
| OT8_P1Rev | ATGTTCACTCCAGCTAGAAG |  |  |
| OT8_P2Fwd | GCAAGCCTGGTGAATGAGGA |  |  |
| OT8_P2Rev | TTCCACAACCCTCCTTTGGC |  |  |
